# Introduction and Goals for the National Children’s Study

**DOI:** 10.3389/fped.2017.00240

**Published:** 2018-02-22

**Authors:** Steven Hirschfeld

**Affiliations:** ^1^Uniformed Services University of the Health Sciences, Bethesda, MD, United States

**Keywords:** child health, child development, longitudinal studies, environmental exposure, growth

## Abstract

The National Children’s Study (NCS) evolved in concept and planning to become an integrated systems based initiative to assess a full spectrum of health and capture the environmental factors and other influences that shape the trajectory of child development. The NCS built on prior work in health assessment, epidemiology, logistics, and methodology in order to address the broad goals of its mandate. To develop the specific methods and tools to conduct a study in multiple environments outside conventional health care delivery clinics the NCS invested in new approaches such as informatics, study operations, and the establishment of a Health Measurements Network to provide accurate, cost effective, and scientifically valid data that would be interoperable with data collected by other longitudinal studies around the world as well as with major national and international health improvement initiatives.

## Introduction and Origins

In 1998, the President’s Task Force on Health Risks and Safety Risks to Children recommended a large study to identify risks associated with broad environmental exposures as a critical first step in addressing environmental risk factors affecting the health and development of children in the United States (US) (1) The National Children’s Study (NCS) was proposed in the year 2000 as part of the Children’s Health Act, Public Law 106-310. Title X Section 1004 of the Children’s Health Act of 2000 authorized the National Institute of Child Health and Human Development (NICHD) to conduct a national longitudinal study of environmental influences on children’s health and development that included three directives.

1. Incorporate behavioral, emotional, education, and contextual consequences to enable a complete assessment of the physical, chemical, biological and psychosocial environmental influences on children’s well being
2. Gather data on environmental influences and outcomes on diverse population for children, which may include the consideration of prenatal exposures.
3. Consider health disparities among children which may include the consideration of prenatal exposures (2).

## Development and Review of Initial NCS Scientific Plan

The NICHD began a consultative and planning period for the NCS in 2001 that included hundreds of scientists. The planning and design of the NCS was informed by existing studies of children’s health and environmental exposures, as well as other related longitudinal studies. Specific input was provided by multiple rounds of independent scientific reviews, expert panels, and comments from the general public received during a series of public meetings.

In 2007 the National Institutes of Health (NIH) requested the Committee on National Statistics of the National Research Council (NRC), in collaboration with the Board on Children, Youth, and Families of the NRC and the Institute of Medicine (IOM) and the IOM Board on Population Health and Public Health Practice, to conduct a review of the research plan for the NCS. The purpose of the review was to assess the scientific rigor of the NCS plan and the extent to which the plan outlined the methods, measures, and collection of data and specimens to maximize the scientific yield of the study.

The NRC/IOM issued a report on September 12, 2008 stating that “the NCS offers an excellent opportunity to examine the effects of environmental influences on child health and development, as well as to explore the complex interactions between genes and environments.” (3). The report further noted: “Nevertheless, there are important weaknesses and shortcomings in the research plan that diminish the study’s expected value below what it might be.”

The IOM report specifically noted five strengths and nine weaknesses:

### Strengths

1. Responsiveness to the Children’s Health Act of 2000.
2. The large number of births to be included.
3. The longitudinal design stretching from before birth until age 21.
4. The many variables to be generated on both outcomes and exposures.
5. The well-designed national probability sample.

### Weaknesses and Shortcomings

1. Absence of an adequate pilot phase.
2. Decentralization of data collection.
3. Inadequacy of plans to maximize response rates and retention rates.
4. Weakness of conceptual model.
5. Weakness of certain data instruments.
6. Insufficient attention to racial, ethnic, and other disparities.
7. Failure to adequately integrate data from medical records.
8. Failure to plan adequately for disclosure of risk to participants.
9. Failure to plan for rapid dissemination of data.

## NCS Vanguard Study

In response to the NRC/IOM report, the NCS developed a pilot phase called the Vanguard Study that was launched in 2009. The Vanguard was to be a pilot at seven locations with the intent to optimize the recruitment methodology and be a lead in for the Main Study. The Vanguard Study seven locations were to be the first locations to be activated with the remaining locations for the larger Main Study to be activated in waves over approximately a 4-year period. This phase of the NCS with seven locations is referred to in this document as the Initial Vanguard Study (4).

In late 2009 a review of the costs and kinetics of recruitment in the initial phase of the Vanguard Study led to the NICHD proposing a new approach to the NCS. The Vanguard Study would no longer be a lead in pilot to optimize the current protocol, but would be a separate study to examine recruitment methodology and systematically pilot a range of assessments at each age group to characterize the feasibility, acceptability and cost of study measures and operations in advance of the Main Study. The Vanguard was reconceived as a dynamic platform to concurrently examine variations on recruitment, content, logistics, and study operations. Due to resource limitations and limited time to resolve multiple questions, the Vanguard evolved to have several substudies and was complex in implementation in that different variations of the protocol were undergoing testing at the same time in different locations. The strategy was that the Vanguard Study would be implemented as a leading parallel study to the planned Main Study, with the Vanguard always testing whatever the next phase of the Main Study would be prior to scale up and implementation. The study protocol would be written in approximately five year intervals to allow preliminary findings and new technologies to inform the next wave of data collection (5).

From 2010 to 2012 the Vanguard Study expanded to 40 locations across the United States with different groups of locations testing different recruitment strategies and different types of assessments. At the same time the NCS initiated a formative research program using most of the 40 locations plus additional collaborators to develop, evaluate and refine technical aspects of data collection.

To specifically address several of the reported weaknesses in the NCS Scientific Plan that the IOM report identified and to consolidate expert input and develop a portfolio and schedule of assessments, the NCS established as a formative research project a Health Measurement Network (HMN). The HMN was a collaborative effort across academic institutions and professional research organizations charged with the development and assessment of tools, instruments, methods, and assays to measure child health and well being. The HMN initially focused on three projects. The first project was development of a living systems perspective on health using the concepts of a series of assets that include energetics, restoration, reproduction, mind, and capabilities. The second project was establishing a typology of related concepts consistent with the living systems health model. The third project was extension of components of the NIH Toolbox for the Assessment of Neurological and Behavioral Function to younger ages. When the NCS operational model of data collection began to shift in 2012 from 36 local contractors to four regional contractors, the HMN also shifted from a formative research initiative to formal integration with study operations.

On March 21, 2013, Congress passed Public Law 113-6, also known as the Consolidated and Further Continuing Appropriations Act of 2013, the fiscal year 2013 funding bill for HHS, including funding amount for NIH, to include the NCS The bill identified that NCS received “Up to $165 million” for 2013. The appropriations language also stated that …” the Director (*of the NIH*) shall contract with the National Academy of Sciences within 60 days of enactment of this Act to appoint an expert Institute of Medicine/National Research Council (IOM/IRC) panel to conduct a comprehensive review and issue a report regarding proposed methodologies for the NCS Main Study, including whether such methodologies are likely to produce scientifically sound results that are generalizable to the United States population and appropriate sub-populations: Provided further, that no contracts shall be awarded for conducting the Main Study until at least 60 days after the IOM/IRC report has been available to the public.” (6). The consequence was that in addition to an approximately 13% budget cut, all work on the planned launch of the Main Study was stopped. The IOM/IRC issued their report in June 2014 and subsequently the NCS was closed out (7, 8).

## Philosophy and Approach

The primary goal of the NCS Main Study was to collect high quality data to enable a complete assessment of the physical, chemical, biological, and psychosocial environmental influences on children’s well being.

The NCS approached the goal applying some key principles

- Frame data collection in a unified schema
- Develop an integrated view of the whole person
- Utilize a life course approach to describe the trajectory of each participant
- Acknowledge and address the complexity of measuring health
- Capture primary data when feasible
- Borrow, leverage, and link to existing data sources
- Develop a visit schedule and process that balances scientific need with pragmatism and attention to the burden on participants and study infrastructure
- Design visit data collection to capture a wide range of exposures and outcomes aligned with the general conceptual model

The application of these principles is outlined in the following sections.

## Frame Data Collection in a Unified Schema

The NCS framed data collection initially through identification of the characteristics of a healthy well-functioning 21-year-old person and then sought to capture the influences and factors that lead to a healthy well-functioning 21-year old. The age of 21 was selected because growth and development, particularly in the central nervous system, typically continue until that age and because the Food and Drug Administration Amendments Act of 2007 defines a child for research purposes to be through age 21 years. In addition, if the NCS were to continue as a national cohort study, all participants in all states would be able to provide consent.

By using the approach of working backwards from the projected end of scheduled data collection to frame the trajectory, the NCS could organize the visit schedule and types of information based on specific information targets. The challenges are to understand how to measure health at different ages and to identify a scientifically credible range of characteristics that describe the health status of a person at different ages.

## Develop an Integrated View of the Whole Person

The NCS considered phenotype as the observable characteristics of the whole person. Observable includes not only visual information, but all types of information that can be captured and assessed. Note that to characterize an individual as an integrated whole person, not all observations may be contemporaneous. Phenotype is the result of the interactions of genotype, environment and development.

For measurement purposes, the NCS established a data model where each individual would have phenotypic + environmental + genotypic data serially collected with all data tagged with time and place and other characteristics. The composite of the phenotypic, environmental, and genotypic collected data combined with linked data from other sources is used to develop an integrated view of the whole person. As a consequence of this structured approach, all data about an individual or the environment can be readily placed into a context.

## Utilize a Life Course Approach to Describe the Trajectory of Each Participant

Life course is defined by Giele and Elder as “a sequence of socially defined events and roles that the individual enacts over time.” (9). Bengston and Allen note that a life course perspective emphasizes the importance of time, context, process, and meaning on human development and family life (10). A life course approach to describing an individual and groups was first applied in a scholarly context in the 1920s and elaborated over the twentieth century. A life course approach individualizes the events and characteristics associated with growth and development across the life span and attempts to explicitly link events and exposures over time and place and between and among people. A life course approach acknowledges the importance of interactions.

## Acknowledge and Address the Complexity of Measuring Health

The NCS developed a health measurement framework grounded in the work of the World Health Organization and others. The framework was further informed by an IOM Workshop in 2004 on measurement of child health.

The fundamental concept was to recognize health as multidimensional and measurable. As in prior schema, the NCS approach did not make the assumption that absence of a disease or diagnosis is sufficient to imply health status, only serving to rule out or deem unlikely those conditions and diseases that were screened for. The NCS health measurement framework was based upon assessment of four dimensions of health, provisionally identified as

- performance—what an individual currently does, typically in daily living
- potential—what an individual could do if challenged, or is latently capable of
- experience—prior exposures and outcomes
- adaptability—changes in phenotypic response or behavior as a result of perceived environmental or contextual changes.

**Figure 1:**
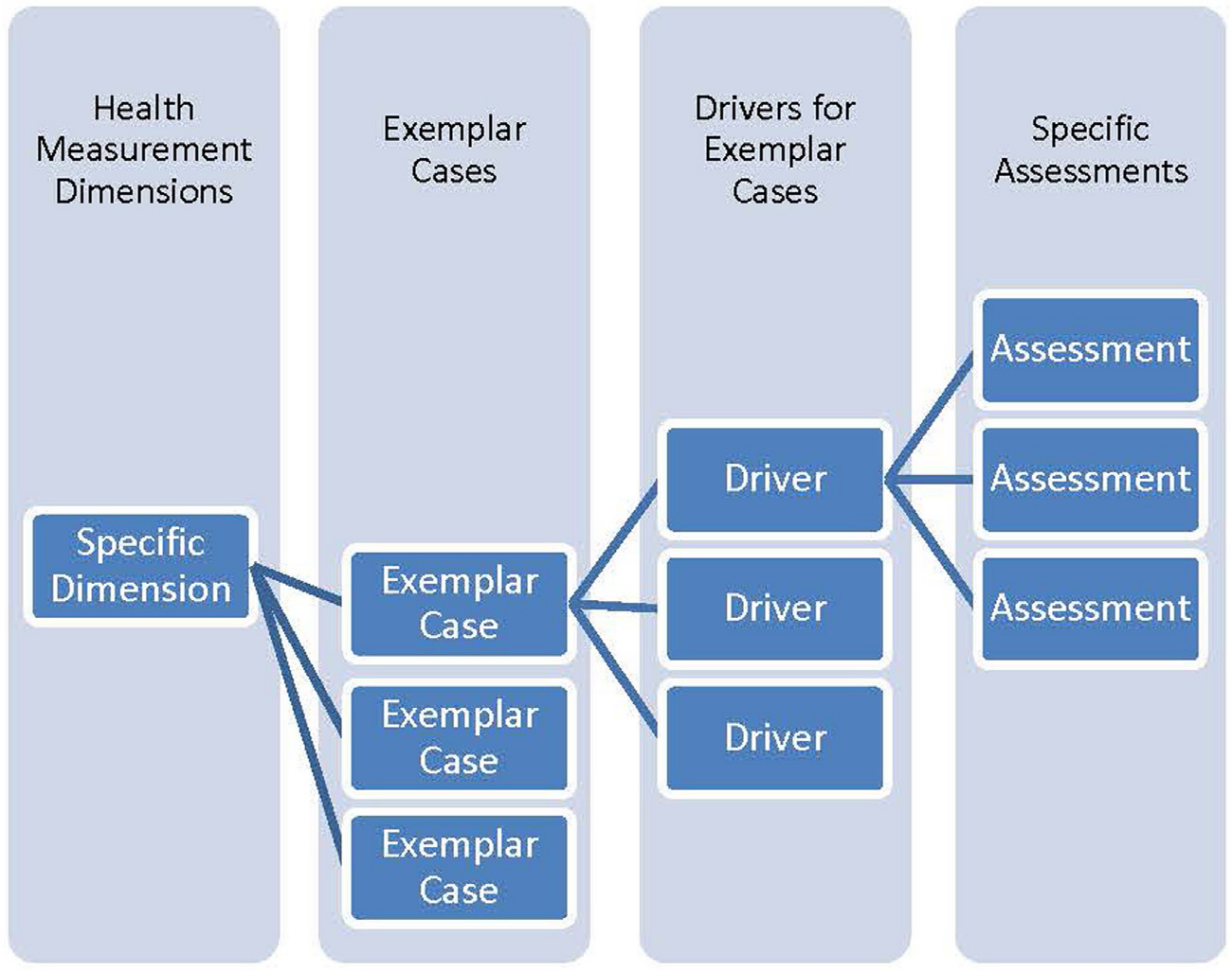
Hierarchy of information from specific assessments to health dimensions. Relationships between health, dimensions, exemplar cases, drivers, and specific assessments is a many to many mapping.

Using the paradigm of the development of a healthy 21 year old, the next step was to identify the characteristics of such a person using exemplar cases for each characteristic. Subsequently, drivers for each exemplar case were identified using a blend of literature references, expert input, and consensus discussion. Lastly, specific assessments were mapped to each driver using a matrix where one axis was a composite listing of all drivers for all the exemplar cases and the other axis was a listing of proposed assessments. In all cases the mappings were many to many. A schematic of the approach is in the following Figure 1.

Reversing the hierarchy, specific assessments inform drivers that in turn inform exemplar cases that in turn contribute to an understanding of the dimensions of health. The composite of all the measurements and dimensions can provide a description of overall health.

Thus from a series of specific assessments, higher order inferences about overall health may be made for an individual or for multiple individuals. These higher order inferences can be plotted versus time and establish a trajectory. The shape of the trajectory can be correlated with phenotypic and other characteristics, environmental exposures, and events. The composite trajectories of multiple people can be used to describe the origins and status of health for populations of interest.

The specifics for the NCS were that the four health dimensions of Performance, Potential, Experience, and Adaptability were mapped to nine exemplar cases of Civic Engagement, Family Relationships and Caregiving, Needs Satisfaction, Peer Relationships, Physical Growth and Metabolism, Physically Active, Readiness for School/Learning/Work, Self-identity/Life Purpose, and Sexual Health. Each exemplar case had a set of associated drivers for different ages. Overall the number of unique drivers was approximately 250. The drivers were mapped to approximately 200 specific assessments, which will be described in further detail below and in related articles. Thus combinations of assessments inform each driver and combinations of drivers inform each exemplar case. The exemplar cases in turn inform each of the overall dimensions of health measurement.

Using such a schema, each assessment can be evaluated and analyzed not only for any single correlations between exposures and outcomes, but for its potential contribution to provide data for the hierarchal matrix consisting of drivers, exemplar cases, and health dimensions. At different ages and stages of development, individual assessments may vary in their relative contributions to the drivers and exemplar cases.

This approach contrasts with an approach used in many other settings where only selected diseases or diagnoses are used as outcomes and correlated with single or small numbers of exposures are correlated. While the NCS remained keenly interested and committed in the developmental and environmental origins of disease, and the proposed NCS data collection system can readily accommodate more conventional analyses, the proposed more robust system provides an opportunity to capture as full a spectrum of outcomes as feasible.

## Capture Primary Data when Feasible

An investment in direct data collection is the principal means to ensure precision and quality. The data and information collected for the NCS was intended to come from participant reports and direct collection including home visits to make direct observations and collect environmental samples in context.

While the NCS was designed to collect information about primary signs, symptoms, and illnesses that may indicate limitations or conditions, it did not commit to a specific nosology to make diagnoses. Classifications and nosologies are constantly undergoing revisions and updates. Capturing primary signs and symptoms is both more precise and flexible in linking exposures, phenotypic information, and events.

The same approach applies to environmental samples where the water output analysis from a home faucet or air particle measurements in sleeping quarters can be more informative than estimates based on geographic coordinates from generic measurements from other databases. In addition, visual and sound recordings can provide data not obtainable by other means regarding movement, coordination, ambulation, speech, ambient noise, and other types of interactions.

## Borrow, Leverage, and Link to Other Data Sources

The NCS recognized that resource constraints of various types would limit data collection. Therefore to achieve the goal of an integrated description of a whole person or groups of people, it is essential to develop a systematic approach to identify and then link data from other sources.

The NCS plan was to incorporate information from multiple other sources to supplement and provide context for direct data collection. One resource the NCS invested in was the construction of a comprehensive database of questions derived from historical and ongoing longitudinal and cross sectional surveys, primarily funded by the federal government. The database was cataloged and tagged with metadata to identify groups of related topics and to facilitate potential analyses by linking results from the NCS with other studies.

Another resource the NCS invested in was the construction of a database linkage project with over 100 federal databases from multiple agencies where single variables can be associated with administrative, environmental, economic, and other data types primarily through temporal and geocoding information.

The NCS collaborated with the National Cancer Institute (NCI) Enterprise Vocabulary Services to harmonize, align, and in some cases develop concepts and terminology specific to maternal and child health and disease. The NCS invested in the establishment of multiple international working groups focused on the topics of perinatology, neonatology, rheumatology, nephrology, infectious diseases, endocrinology, hematology and oncology, and a comprehensive glossary for pediatric adverse events. All the concepts and terms were integrated into the NCI Metathesaurus. The terminology is currently available for public access through the NCI Term Browser^1^ and the NCI ftp site.^2^ Through direct collaboration with the Medical Dictionary for Regulatory Activities (MedDRA), many of the terms have also been incorporated into MedDRA.

The NCS co-sponsored an international conference on terminology harmonization to receive input and also increase awareness of the value of harmonized terminology to improve precision in research, health care delivery, epidemiology, and analyses. See http://www.nichd.nih.gov/about/meetings/2012/Pages/092112.aspx, which was videocast in http://videocast.nih.gov/Summary.asp?file=17588. Participants included electronic health record vendors, the National Library of Medicine, professional organizations, and academic researchers.

The NCS collaborated with the NIH Toolbox for the Assessment of Neurological and Behavioral Function. The NIH Toolbox is designed to be a set of short assessments to measure emotional, cognitive, sensory, and motor function in children and adults ages 3–85 (11). These assessments intend to evaluate function over time and across developmental stages; a necessary requirement for any longitudinal assessment. The NCS invested in extending the range of measures, developing new measures, and adapting the NIH Toolbox from a server based data model to a portable capability using the iPad.

The NCS also collaborated with the NIH Patient-Reported Outcome Measurement Information System to support additional development of pediatric outcome measures (12).

Recognizing the power and the potential for metadata to link and characterize data from multiple sources, the NCS organized an international conference on metadata^3^ and, based on input from that meeting as well as other sources, began compiling metadata standards and a repository for metadata to generate a consistent approach to link, retrieve, associate, and analyze data.

Additional linkage sources the NCS intended to include were medical records which may contain information about diagnoses, treatment plans and outcomes. Further sources were to be school records, weather records, air particulate matter levels, pollen counts, social and political events, crime, violence, access to fresh food, green space, recreation, cultural and sport activities, and other types of exposures. The NCS held a series of discussions with several health provider networks, the i2b initiative (13) and others to develop cost effective mechanisms to share electronic medical record data, evolving to a model where the NCS would designate approximately 200 variables for any NCS participants that would be transmitted to a neutral secure data environment at fixed intervals. The NCS could then process the variables, request clarifications if needed, and subsequently integrate the data with other participant level data.

The NCS participated in discussions, and in some cases collaboration, with other studies to align data collection methods, variables, and data sharing. One was the National Health and Nutrition Examination Survey which resulted in an ongoing exchange of instruments, methods, and processes. The NCS also interacted with birth cohort studies conducted in other countries. To that end, the NCS participated in the Environment and Child Health International Birth Cohort Group comprised of large birth cohort studies from China, France, Germany, Japan, and the US (14). The group met regularly to share expertise, current study practices, and projects to harmonize study visit procedures and processes. The NCS also maintained less structured contact with several longitudinal birth cohort study investigators in locations such as Australia, Canada, New Zealand, South Korea, and the United Kingdom.

The logical framework was that each participant would have a multidimensional data array organized around the primary collected data, biospecimens, and environmental samples consisting of extant data related to the individual (such as electronic medical records, school records, data from other studies an individual may participate in) plus generic administrative, environmental, social, political, judicial, cultural, and other data related to time and place. The intersection of all these data was intended to convey opportunities to future analysts that may not be currently conceivable.

All of these resources and collaborations were intended to be publicly available as part of a larger plan for the NCS to function as a service center for other studies to provide harmonized content, linkages, and metadata tags. Currently, the NIH Toolbox, NCI Term Browser, and MedDRA include the NCS supported material in each of their respective repositories.

## Develop a Visit Schedule and Process That Balances Scientific Need with Pragmatism and Attention to the Burden on Participants and Study Infrastructure

The NCS visit schedule as developed prior to the initial IOM review had 12 visits, five were home visits and seven were clinic based. The schedule is summarized in the following Table 1.

**Table 1:** National Children’s Study visit schedule as proposed in 2007.

Schedule	Home visit	Clinic visit
First trimester	x	
Second trimester with ultrasound		x
Third trimester	x	
Delivery		x
6 months	x	
12 months	x	
3 years		x
5 years		x
8 years	x	
12 years		x
16 years	x	
20 years		x

*Letter “x” denotes the visit is scheduled*.

The NCS re-evaluted the distribution of visits and used a general principle of capturing data where the largest knowledge gaps and rapid changes occurred. In addition, the NCS was informed by the recommended visit schedule for the Bright Futures program.

In 1994, the federal Maternal and Child Health Bureau of the Health Resources and Services Administration (HRSA) and the Healthcare Financing Administration, which is now part of the Center for Medicaid and Medicare Services, published the first edition of *Bright Futures: Guidelines for Health Supervision of Infants, Children, and Adolescents* (15). A second edition followed in the year 2000, and in 2001, HRSA awarded a cooperative agreement to the American Academy of Pediatrics to develop a third edition that was released in 2008 (16). The Bright Futures program goals are to provide a framework for health-care services delivery that is prevention based, family focused, and developmentally oriented. The Bright Futures program is implemented through health-care service providers.

Bright Futures is organized around 10 themes that are promoting:

- family support
- child development
- mental health
- healthy weight
- healthy nutrition
- physical activity
- oral health
- healthy sexual development and sexuality
- safety and injury prevention
- community relationships and resources

Note the similarities of the Bright Futures themes to the topics of the Exemplar Cases used to describe health dimensions for the NCS. The NCS topics were civic engagement, family relationships and caregiving, needs satisfaction, peer relationships, physical growth and metabolism, physically active, readiness for school/learning/work, self-identity/life purpose, and sexual health.

**Figure 2:**
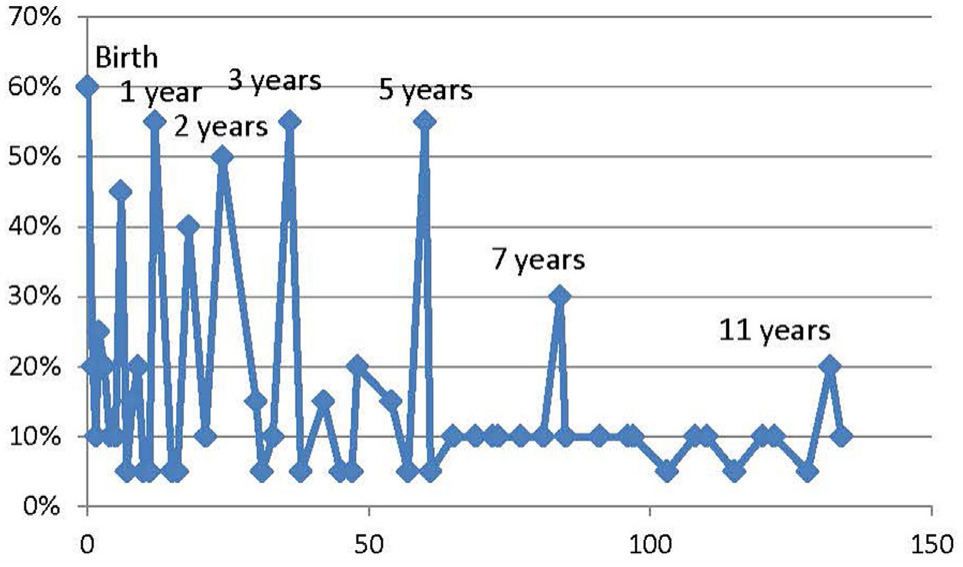
Distribution of Scheduled Study Visits among 20 longitudinal birth cohorts. Consensus assessments points among multiple international cohort studies result in some consistent patterns.

Another reference frame for determining a visit schedule is the schedule from other longitudinal birth cohorts. Through a combination of literature review, study specific internet sites, and personal communications, the NCS obtained visit schedules for 20 longitudinal studies that examined exposures and outcomes. Of the 20, 11 (55%) had between one and four pregnancy visits scheduled. Examining the number and distribution of visits over the first 11 years, 13 (65%) of the studies had no more scheduled visits after that age. The average number of scheduled assessments across all studies between birth and 11 years was seven. The distribution of scheduled visits between birth and 11 years is shown in the following graph, Figure 2, with the per cent of studies scheduling a visit at a given age on the ordinate and the age of the child in months on the abscissa. Additional details regarding the cited studies are provided in the Supplementary Material.

Using the information from the other studies plus the Bright Futures model plus input from subject matter experts plus the focus on increasing assessment frequency during the period of most rapid growth and development, the NCS proposed to evaluate a new schedule for the Vanguard Study in 2010. The revised schedule for the NCS Vanguard study is shown in Table 2.

**Table 2:** Revised National Children’s Study Vanguard Study visit schedule from 2010.

Stage	Schedule	Home visit	Remote data collection
Prenatal and infancy	Pregnancy <20 weeks	x	
	Pregnancy >20 weeks	x	
	Delivery	In-person visit at birth facility	
	3 months		x
	6 months	x	
	9 months		x
	12 months/1 year	x	
Early childhood	18 months		x
	24 months/2 years	x	
	30 months		x
	36 months/3 years	x	
	42 months		x
	48 months/4 years	x	
	54 months		x
	60 months/5 years	x	
Youth	7 years	x	
	9 years	x	
	11 years	x	
	13 years	x	
	15 years	x	
	17 years	x	
	17 years	x	
	19 years	x	
	21 years	x	

*Letter “x” denotes the visit is scheduled*.

The new Vanguard study now had 24 scheduled visits of which 17 were home visits, 6 were remote visits and the delivery or birth visit was in whatever facility the birth occurred. The increased number of visits, particularly during the early years, was intended to address knowledge gaps during a period of rapid change and also build the trusting relationship between the families and the NCS that is critical to ensure continuity and minimize attrition.

The frequency of the visit schedule also had the advantage of distributing assessments across visits to decrease the expectations and burden on any specific visit. Some of the home visits in the initial Vanguard experience were empirically multi-hour encounters with attendant burden on participants and study personnel.

The NCS decision to focus on home visits was based on two principles. One is that direct observation and of the child and family in the environment where they live plus the opportunity to collect samples from that environment forms a critical component of the data model of placing all observations in an environmental context. The other is to maintain as much end to end quality control over data collection as feasible. Challenges with clinic visits are the need for training and the potential for highly variable quality if a given provider collects data on only one or a few participants per year combined with the need for timely and accurate transmission of data from the local system to the NCS central data repository.

The NCS also explored remote data collection using internet connected devices and identified several challenges. A primary determinant was that in 2012 not all participants were likely to have access to a secure remote data collection for electronic transfer. Providing all participants with smartphones or connected tablets was prohibitive with regard to cost for the purchase and the programming involved, plus the additional costs of instituting a support and hardware replacement plan. Other studies that employed device-based remote data collection often included as an eligibility criterion that participants supply their own device and would receive compensatory time on their data plan for transmitting study-related information, but this was not feasible for the NCS. In addition, the NCS encountered cybersecurity concerns for some of the then currently available device operating systems with the additional complication that the data could be classified as personal health information and would need robust technical solutions. Consequently, given the costs and complications, the NCS deferred implementation of device-based remote data collection pending further evolution of acceptable data platforms, secure transmission protocols, and changes in hardware and service pricing.

Ultimately the feasibility of the visit schedule is the intersection between scientific need and feasibility. During the Vanguard Study, the NCS analyzed the costs, burden, content, and modality of visits administered plus adjustments for when expected events and capabilities are likely to occur during growth and development plus newer developments on linking and combining data from external sources to propose the schedule as in Table 3. The total number of data collection opportunities is 31 with 17 in-person home visits and 14 remote data collections. While the number of in-person home visits in the 2014 version remains the same as the 2012 Vanguard Study schedule, the timing and spacing is shifted in the earlier years. The number of remote visits increased to leverage emerging technology for secure remote data collection.

**Table 3:** National Children’s Study Proposed Visit Schedule from 2014.

Visit	Home	Telephonic remote data collection
Pregnancy visit 1 (<20 weeks)	x	
Pregnancy visit 2 (>20 weeks)	x	
Birth	In-person visit at birth facility	
4 months		x
8 months	x	
11 months		x
14 months for child and preconception for future children if the mother is not pregnant or pregnancy visit 1 if the mother is pregnant	x	
18 months		x
21 months	x	
30 months		x
3 years	x	
42 months		x
4 years	x	
54 months		x
5 years	x	
6 years		x
7 years	x	
8 years		x
9 years	x	
10 years		x
11 years	x	
12 years		x
13 years	x	
14 years		x
15 years	x	
16 years		x
17 years	x	
18 years		x
19 years	x	
20 years		x
21 years	x	

*Letter “x” denotes the visit is scheduled*.

At the 14-month child home visit, the mother will be assessed either as a preconception data collection should she give birth to additional children while the NCS is still recruiting or as a first pregnancy visit if she is already pregnant. Fourteen months was selected for this particular maternal assessment for several reasons. Generally breast feeding is completed by that time. Furthermore, published data from the United States and other countries indicate the average interbirth interval between first and second born children is approximately 30 months with conception generally occurring between 18 and 23 months following birth of the first child. Approximately 60% of US. mothers have a second child. The prospective collection of preconception data can facilitate better understanding of prenatal factors and exposures that influence birth outcomes and childhood growth and development. While the proposed NCS plan will not capture preconception data on women who are nulliparous, the opportunity for data capture on thousands of women who well may have additional children is available at modest cost.

## Design Visit Data Collection to Capture a Wide Range of Exposures and Outcomes Aligned with the General Conceptual Model

A few principles guided the selection of what data to collect. One was adherence to the study goals. The NCS mandate to “incorporate behavioral, emotional, education, and contextual consequences to enable a complete assessment of the physical, chemical, biological, and psychosocial environmental influences on children’s well being” dictates that the content be framed by a need to understand growth and development across a broad spectrum of outcomes. The spectrum of outcomes ranges from uncommon debilitating conditions to frequent phenotypic states to uncommon and extraordinary capabilities. Anticipating a broad range of outcomes is a mechanism to permit analyses that may not be anticipated during the design and implementation phase of the NCS. Potential analyses may address relationships that may not currently be possible to imagine. Thus, the rationale for collecting any specimen and making any assessment is that the resulting data can be used for an analysis that will inform the study goals of a complete assessment of the influences on children’s well being.

A second principle was that the process of data collection must be efficient and parsimonious with limits on the length of each assessment and the total encounter burden. The NCS approach to framing data collection was developing sufficiently precise and specific content to support complex analyses while maintaining respect for participants’ time and remaining cost effective. As initially planned, some of the home visits empirically took more than 6 h, required two people and two back packs with approximately 70 lbs of equipment and monitors.

A third principle was to build upon and link with visit schedules and content from other contexts. Utilizing portions of the recommended assessments from the HRSA-American Academy of Pediatric Bright Futures program and from the Centers of Disease Control and Prevention National Center for Birth Defects and Developmental Disabilities (16), the NCS included assessments in the domains of social and emotional, cognitive, movement and physical development, and language and communication. In addition, if triggered by other information, a disability score based on the World Health Organization International Classification of Functioning, Disability, and Health for Children and Youth can be recorded (17) While the stand alone version of the classification tool for children and youth is still available and in use, the World Health Organization began a consolidation process in 2010 of the general classification with the version for children and youth.

To consolidate expert input and develop a portfolio and schedule of assessments, the NCS established, as noted above, a HMN. Following a formative research phase, the HMN became integrated with Vanguard Study operations. Operationally, the HMN consisted of subject matter experts primarily from academic institutions organized into domain teams with each team having a roster of one lead, and four to six subject matter experts. Each team met regularly by teleconference and occasional face to face meetings. All the team leads plus additional consultants and NCS Program Office staff met regularly as a Steering Committee by teleconference to coordinate approaches and everyone met face to face for a 2-day meeting at least once a year.

The domain teams were cognition, environment, life course, motor, physical health, sensory, social–emotional–behavioral, statistics, and translatability–multicultural–accessibility which included disabilities and health disparities based on several criteria. Each team began with the premise of tracking and assessing the relevant domains from as early as pregnancy to age 21 years in alignment with the study goals. The teams surveyed available assessment options, identified gaps at particular ages, and if need be, proposed and developed new assessments to address any gaps. The activities were thus a blend of survey, gap analysis, formative research, and expert recommendations. As the teams identified and refined their recommendations, the Steering Committee provided input on integrating the recommendations, prioritizing them, and assigning assessments to scheduled visits, balancing total visit length with age appropriateness of the assessment.

All proposed visits were integrated with a core questionnaire and assessments designed to provide continuity and key age appropriate information across all visits.

In addition to the HMN input and the core questionnaire, the NCS developed a comprehensive database of federally sponsored surveys from which it could draw relevant modules. All items were extracted as modules that contained all relevant material from the initial surveys and tagged with metadata regarding general topic, source, intent, scoring, references, and other information. Recognizing that questions are often designed as a series and extracting an isolated question from the series can affect the validity and interpretation of the responses, linked and nested questions were tagged appropriately.

## Content Development Process

In addition to emphasizing specific content, the NCS analyzed, invested in, and tracked methods to improve the content development process. The existing timeline when NCS operations began in 2009 for implementing a new item into the field taking into account selection, integration into a visit, and programming into study visit instruments with quality control steps was 12 months. Following implementation of a revised workflow process and running some activities in parallel reduced the timeline to 4–6 months depending upon the precise nature and content of the item. Key steps in improving the process were identification of handoffs and bottlenecks, setting performance and quality assurance standards, develop of visit choreography in modules, and the timing and organization of the overall workflow process.

**Figure 3:**
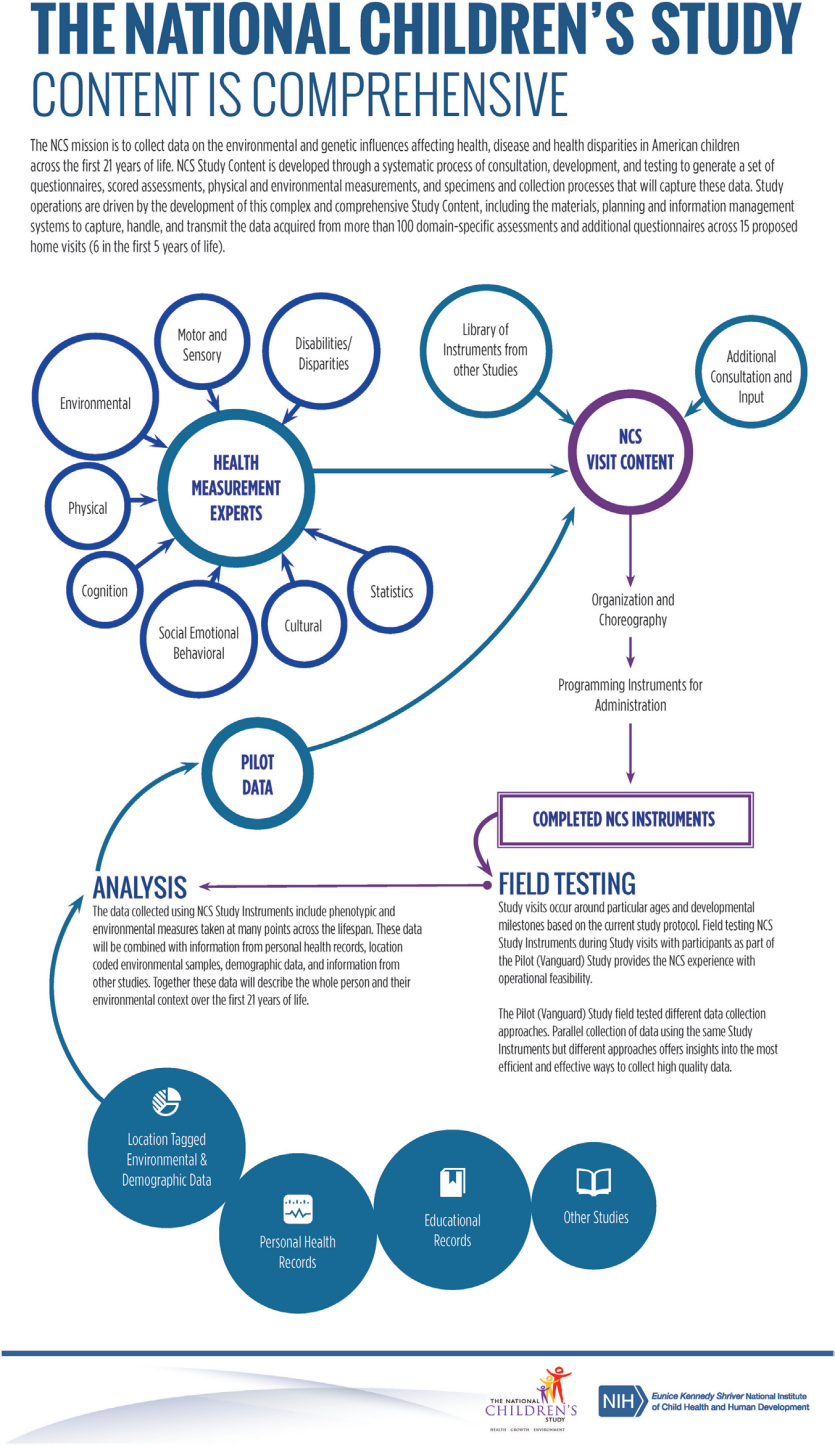
NCS Content Development process. The proposed relations hips and workflow process to integrate material from multiple sources into an integrated framework.

A diagrammatic summary of the NCS content development process is in Figure 3.

The NCS plan was to field test all assessment proposals with variations in the Vanguard Study to document the timing estimates, feasibility, acceptability, and costs. Thus, the Vanguard Study would remain an ongoing incubator or laboratory to provide the data necessary for scale up, resource planning, and project management for the Main Study.

The proposed matrix of visit schedule and planned assessments from 2014 had approximately 200 items distributed over the 14 post-birth scheduled visits. Each domain category had several subdomains and each subdomain. A summary of the general framework is in Table 4. Listed are examples of major domains with the number of subdomains that have specific assessments associated with them. For each scheduled visit the number of assessments for a given domain is listed with the understanding that a single subdomain may contain an assessment that has multiple items. Thus, the number “one” may represent a multi-item assessment. The last column displays the mean number of assessments for the listed domain per study visit with a range of approximately 2–15. The last row displays the total number of assessments scheduled for a particular visit with a range of approximately 40–100 and a mean of 60. All the assessments are timed so that the total length of a given home visit does not exceed 3 h including environmental sample collection.

**Table 4:** Distribution of assessments across post-birth National Children’s Study visits.

Domain	Number of subdomains	8 months	14 months	21 months	3 years	4 years	5 years	7 years	9 years	11 years	13 years	15 years	17 years	19 years	21 years	Mean
Cognition	40	14	13	11	7	3	9	10	7	4	8	4	9	5	6	7.9
Sensory	16	5	2	4	3	4	6	5	4	4	5	2	4	3	4	3.9
Motor	11	1	1	1	3	4	3	5	4	4	1	1	0	2	2	2.3
Social–emotional–behavior	73	6	3	4	7	0	2	23	28	27	23	2	43	22	7	14.1
Physical	22	11	12	13	15	14	16	16	18	17	17	16	18	16	18	15.5
Environment	40	20	8	19	8	9	6	7	26	9	25	15	28	10	25	15.4
General		1	1	1	1	1	1	1	1	1	1	1	1	1	1	
Total	202	58	40	53	44	35	43	67	88	66	80	41	103	59	63	60.0

## Modes of Study Visit Administration

Each study visit was designed to be conducted across multiple modes (in person, telephone, mail, internet); methods of administration (interviewer-administered; self-administered; computer assisted versus paper-based); and locations. For instance, questionnaires addressing sensitive topics may best be administered by telephone or *via* mailed surveys or secure remote data connection, as in-person administration may increase bias or inhibit information sharing. Study visits that require measurements, observations, sample, or specimen collection by a trained data collector must be conducted in person.

The NCS Vanguard Study was framed as a learning environment and not as a platform for capturing definitive information regarding exposure and outcome relationships. The NCS Vanguard Study took an approach that for each proposed assessment, a sufficient number of instances of the assessment would be attempted to determine the feasibility, acceptability, and costs associated with administration. Thus, each proposed assessment was not necessarily planned to be administered to the entire enrolled study population, but rather was evaluated on a periodic basis until sufficient information was collected to permit analysis of the performance characteristics. Proposed assessments that had sufficient data to analyze were retired from active administration and replaced with other assessments.

In addition, variants on study procedures were explored concurrently. For example, different recruitment strategies different data collection procedures, different informatics platforms, and different assessments were implemented at different locations. While operationally complex, the concurrent implementation allowed direct comparisons and was more time and resource efficient than consecutive testing. No single contractor was responsible for more than one methodology or approach, but because multiple contractors were involved, the opportunity for implementation of multiple concurrent approaches became feasible. As an example, one contractor used mobile vans equipped with relevant instrumentation to conduct part of the home visits in a specially designed environment while other contractors used standard personal vehicles to transport needed equipment, supplies, and instruments, and conducted all the testing in the home.

## Questionnaires

Questionnaires were designed with the input of subject matter experts and the greater NCS community of contractors and stakeholders. The NCS data collection plan included a Core Questionnaire to assess constructs that are important to measure repeatedly as a child ages. It is designed to collect core data longitudinally beginning when the enrolled child is 6 months old. Some examples of constructs included in the core questionnaire are: housing characteristics and composition, pesticide applications, childcare arrangements, health insurance and health-care utilization and access, well-child care and immunizations, emergency room visits and hospitalizations, medications, sleeping patterns as well as additional topics. Along with the core questionnaire, participants will complete age-specific or special topic modules. The age-specific modules include questions that measure constructs particularly relevant the corresponding age, based on known developmental milestones.

In general, participants were asked to complete interviews and self-administered questionnaires on a variety of topics including:

- demographics
- current pregnancy history
- reproductive history
- medical conditions
- health behaviors
- doctor visits
- medicines and supplements
- housing characteristics
- pesticides, product use
- occupation, hobbies
- depression, stress, and mental health
- social support
- diet, exercise, and metabolism
- time and activity

## Informatics

No available informatics platform could address the requirements of the NCS. Consequently, the NCS developed a program to systematically analyze informatics platforms. Cognizant of the duration of the study, the NCS established several general conditions including that the general platform be non-proprietary, conformed with international standards regarding data formats and transmission protocols, system security was consistent with federal government standards, the architecture was modular in design to allow substitution of components without changing the entire platform, that the platform be interoperable with other data resources, and that the system would capture operational or process data as well as protocol directed assessments.

During the course of the Vanguard Study, several systems were tested concurrently with a structured process for evaluation. Over the course of the Vanguard Study, the NCS systematically evaluated a series of options with progressive learning and improvement at each cycle. When the Vanguard Study was terminated, the NCS was in the process of consolidating three systems into a single platform.

The final Vanguard Study IMS would serve as the conceptual model for the development of the Main Study IMS to provide administrative, computational, reporting, and telecommunications support for the NCS. The Main Study informatics system requirements are to capture data based on the overall study objectives of:

1. recruit and retain a diverse national probability cohort of children born in the United States over a 4-year period
2. systematically collect data about exposures from prenatal to age 21 years from the cohort
3. systematically assess outcomes, including health disparities, from the cohort guided by a multidimensional model of health with targets and methods to reduce the variance for potential exposure–outcome relationships
4. establish a data repository and specimen archive to support analyses of potential exposure–outcome relationships
5. establish mechanisms to share data, data sets, biospecimens, and environmental samples to a broad spectrum of investigators and stakeholders

The system will thus require a case-management module, a survey module capable of supporting in-person and remote data collection, biospecimen and environmental sample collection, and tracking module and service modules to track performance, costs, and quality.

The estimated number of variables is about 100 for case management, approximately 4,000 to cover the projected 280 data content domains, approximately 100 for specimen and sample management, and approximately 500 to cover the service modules or about 5,000 in total. The data types will be text, numbers, images, videos, and sound recordings. For 100,000 participants with 15–17 scheduled data collections and an estimated 300 variables for each visit, the estimated number of data points would be approximately 500 million over the course of the study. Video and photographic storage is estimated at 45 petabytes. Nucleic acid sequences will not be stored by the NCS, just DNA and RNA samples. Sequence data will be linked to other resources.

The NCS data systems were designed to comply with the Data Document Initiative and the Clinical Data Interchange Standards Consortium data models and standards (18, 19). The NCS collaborated with the NCI Enterprise Vocabulary Services for terminology. The NCS developed child health oriented terminology to fill gaps in the larger international resources such as the Medical Dictionary for Regulatory Affairs (MedDRA), Systematized Nomenclature for Medicine-Clinical Terms (SNOMED-CT) and the International Classification of Disease. The terminology developed by the NCS was coordinated with international standards organizations.

## Alignment with Other Maternal and Child Health Surveys

The importance of a healthy population is the topic of multiple national and international initiatives. In September 2000 the United Nations held a Millennium Summit and launched the Millennium Development Goals by issuing declaration 55/2 with a target of the year 2015 (20) They were:

1. to eradicate extreme poverty and hunger
2. to achieve universal primary education
3. to promote gender equality and empower women
4. to reduce child mortality
5. to improve maternal health
6. to whom it may concern: combat HIV/AIDS, malaria, and other diseases
7. to ensure environmental sustainability
8. to develop a global partnership for development

Of the eight goals, the first six can be considered directly related to child health and have subgoals, as summarized in Table 5.

**Table 5:** United Nations Millennium Goals related to child health assessment.

Goal	Subgoal	Metric
Goal 1: eradicate extreme poverty and hunger	Target 1A: halve, between 1990 and 2015, the proportion of people living on less than $1.25 a day	Poverty gap ratio [incidence × depth of poverty]
		Share of poorest quintile in national consumption
	Target 1B: achieve decent employment for women, men, and young people	GDP growth per employed person
		Employment rate
		Proportion of employed population below $1.25 per day (PPP values)
		Proportion of family-based workers in employed population
	Target 1C: halve, between 1990 and 2015, the proportion of people who suffer from hunger	Prevalence of underweight children under 5 years of age
		Proportion of population below minimum level of dietary energy consumption

Goal 2: achieve universal primary education	Target 2A: By 2015, all children can complete a full course of primary schooling, girls and boys	Enrollment in primary education
		Completion of primary education

Goal 3: promote gender equality and empower women	Target 3A: Eliminate gender disparity in primary and secondary education preferably by 2005 and at all levels by 2015	Ratios of girls to boys in primary, secondary, and tertiary education
		Share of women in wage employment in the non-agricultural sector
		Proportion of seats held by women in national parliament

Goal 4: reduce child mortality rates	Target 4A: Reduce by two-thirds, between 1990 and 2015, the under-5 mortality rate	Under-5 mortality rate
		Infant (under 1) mortality rate
		Proportion of 1-year-old children immunized against measles

Goal 5: improve maternal health	Target 5A: reduce by three-quarters, between 1990 and 2015, the maternal mortality ratio	Maternal mortality ratio
		Proportion of births attended by skilled health personnel
	Target 5B: achieve, by 2015, universal access to reproductive health	Contraceptive prevalence rate
		Adolescent birth rate
		Antenatal care coverage
		Unmet need for family planning

Goal 6: Combat HIV/AIDS, malaria, and other diseases	Target 6A: have halted by 2015 and begun to reverse the spread of HIV/AIDS	HIV prevalence among population aged 15–24 years
		Condom use at last high-risk sex
		Proportion of population aged 15–24 years with comprehensive correct knowledge of HIV/AIDS
	Target 6B: achieve, by 2010, universal access to treatment for HIV/AIDS for all those who need it	Proportion of population with advanced HIV infection with access to antiretroviral drugs
	Target 6C: have halted by 2015 and begun to reverse the incidence of malaria and other major diseases	Prevalence and death rates associated with malaria
		Proportion of children under five sleeping under insecticide-treated bed nets
		Proportion of children under five with fever who are treated with appropriate antimalarial drugs
		Incidence, prevalence, and death rates associated with tuberculosis
		Proportion of tuberculosis cases detected and cured under Directly Observed Treatment Short Course

Also in the year 2000, the Save the Children Foundation began publication of an annual report on the State of the World’s Mothers ranking mother’s and children’s well being in over 100 countries (21). The report captures five metrics for each of the countries, ranks each country for each category, and then for a given country takes the average ranking of all categories with each category having equal weight, for an overall index. All countries are then ranked based on the overall index. The five metrics are summarized in the following Table 6.

**Table 6:** Mother’s Index metrics used by Save the Children Foundation.

Maternal health	Lifetime risk of maternal death (1 in number stated)
Children’s well being	Under 5 mortality rate (per 1,000 live births)
Educational status	Expected number of years of formal schooling
Economic status	Gross national income per capita (current US$)
Political status	Participation of women in national government (% seats held by women)

In the United States, the Annie E. Casey Foundation began publication of an annual data book known as Kids Count in 1990 and subsequently an online data center. Sixteen categories are tabulated, which are summarized in the following Table 7 (22).

**Table 7:** Overall well being of children in the United States assessed by Kids Count.

Category	Metric
Economic well being	Children in poverty
	Children whose parents lack secure employment
	Children living in households with a high housing cost burden
	Teens not in school and not working
Education	Children not attending preschool
	Fourth graders not proficient in reading
	Eighth graders not proficient in math
	High school students not graduating on time
Health	Low birth weight babies
	Children without health insurance
	Child and teen deaths per 100,000
	Teens who abuse alcohol or drugs
Family and community	Children in single parent families
	Children in families where the household head lacks a high school diploma
	Children living in high poverty areas
	Teen births per thousand

In the year 2010, the United States Department of Health and Human Services launched the Healthy People 2020 that targets 26 goals summarized in the following Table 8 (23).

**Table 8:** United States Healthy People 2020 Metrics.

Category	Metric
Access to health services	Persons with medical insurance
	Persons with a usual primary care provider
Clinical preventive services	Adults who receive a colorectal cancer screening based on the most recent guidelines
	Adults with hypertension whose blood pressure is under control
	Persons with diagnosed diabetes whose A1c value is >9%
	Children aged 19–35 months who receive the recommended doses of DTaP, polio, MMR, Hib, hepatitis B, varicella, and PCV vaccines
Environmental quality	Air quality index exceeding 100
	Children exposed to second-hand smoke
Injury and violence	Fatal injuries
	Homicides
Maternal, infant, and child health	All Infant deaths
	Total preterm live births
Mental health	Suicides
	Adolescents who experience major depressive episodes
Nutrition, physical activity, and obesity	Adults who meet current Federal physical activity guidelines for aerobic physical activity and muscle-strengthening activity
	Adults who are obese
	Obesity among children and adolescents
	Total vegetable intake for persons aged 2 years and older
Oral health	Children, adolescents, and adults who visited the dentist in the past year
Reproductive and sexual health	Sexually active females aged 15–44 years who received reproductive health services in the past 12 months
	Knowledge of serostatus among HIV-positive persons
Social determinants	Students who graduate with a regular diploma 4 years after starting 9th grade
Substance abuse	Adolescents using alcohol or any illicit drugs during the past 30 days
	Adults engaging in binge drinking during the past 30 days
Tobacco	Adults who are current cigarette smokers
	Adolescents who smoked cigarettes in the past 30 days

The United Nations and United States target programs, combined with the status reports generated from the Annie E. Casey and Save the Children foundations, use a total of 78 metrics. These high level measures are general surrogates for a wide range of other characteristics and outcomes that reflect policy, environment, economic, and social conditions. The measures can be cataloged into 15 categories with the frequency of each metric among the surveys listed with each category in the following Table 9.

**Table 9:** Distribution of outcome metrics among selected major programs and reports.

Category	Number of metrics
Economic	13
Mortality	12
Access to health-care services	11
Education	10
Preventive	8
Alcohol and tobacco use	5
Birth related	4
Chronic diseases	3
Nutrition	3
Physical environment	2
Obesity	2
Political engagement of women	2
Mental health	1
Physical activity	1
Social environment	1

The NCS planned data collection provided information for all these categories and was designed to provide a link from the individual to the broad metrics used to characterize child health on a population basis.

## Summary and Conclusion

The planning and design for the NCS main study was informed by:

1. completed and ongoing studies of children’s health and environmental exposuresas well as other related longitudinal studies
2. the empirical experience of the Vanguard Study
3. the review and comments provided by multiple rounds of independent scientific reviews and expert panels as well as by comments from the general public received during meetings and Requests for Information

The NCS took a long-term perspective and had to develop a conceptual framework, recruitment approaches, data collection content and methods, an informatics platform, and analytic resources in order to meet its goals and anticipate that conditions, resources, and priorities will continue to change over the next quarter century. Operationally, the NCS also took a systems approach and concurrently strove to become a learning environment where data about the process as well as the content would be collected to enable informed decision-making. The NCS was the first major study to specifically integrate the Collaborative Improvement Network approach into its operations.

The NCS took a systems approach by linking a scientific and metric framework with a layered approach to content development and data collection. A layered approach means that similar activities are clustered and interconnected and then the clusters or layers are in turn interconnected. Input came from a range of sources, in many cases though identification and selection of pre-existing material and in some cases identification of a need for something new that is relevant to a context and population of interest. In addition to evaluating content, the relationship of any proposed content to other content, alignment with the proposed health measurement framework, feasibility, and cost were all factors to consider and integrate. For example, if a proposed assessment was informative for multiple drivers and could inform several exemplar cases in the conceptual framework, the analytic utility of that assessment would be a factor in determining if and how to use it.

The NCS developed a workflow model of assembling information, gap analysis, formative research, testing, validation, and scale up in the Vanguard Study, and then planned transition and further scale up to the Main Study. That workflow process could not only serve the NCS but benefit other studies.

A blend of collected data plus selected administrative data from multiple sources plus health and education records can provide a comprehensive view. What data sources are plausible to provide data in such a model, how the data can be integrated, and what resources and tools are needed will be the operational key. Use of data standards, metadata linkages, and new business models for data use and reuse are essential to make the model functional.

To address the broad mandate, the NCS developed resources to effectively and comprehensively identify and capture potentially relevant material from other studies as well as perform gap analyses and support the development and testing of new material. In selected cases, the process was done collaboratively such as the partnerships with other international studies.

The goal of emphasizing process and integrated development was for the Vanguard Study to inform and become a resource for more than the NCS Main Study. Any study that enrolled children could benefit from the knowledge bases, the techniques, the survey questions, the developmental and functional assessments, and the workflow processes and informatics the NCS invested in. The NCS was thus positioned to become a content and process resource for child health research in general. The elements developed as core elements for the NCS were developed based on utility, parsimony, and information content. In such a business model, the next generation of child health research studies could draw on the NCS for specific high quality tested and validated components and compare results to the large, diverse, NCS cohort in order to normalize and interpret outcomes. Thus, the NCS would become a reference resource for multiple studies using relevant and appropriate subsets or combinations of NCS validated instruments and assessments across the child health age spectrum.

The following articles in this volume elaborate on the general framework beginning with

- conceptual model—framing and developing the life course based multidimensional conceptual model for measurement
- considerations for data collection—an overview of the statistical and questionnaire expectations followed by specific considerations regarding need to accommodate people with accessibility and other needs and address linguistic diversity
- multicultural and health disparities—an initial conceptual approach followed by considerations for implementation
- assessment of motor, sensory, and physical domains—the process and recommendations for assessing these domains
- assessment of cognition—the process and recommendation for assessing cognition
- assessment of social–emotional–behavior domains—the process and recommendations for assessing these domains
- environment—the process and recommendations for capturing and assessing the complex interplay of exposures

## Author Contributions

SH conceived, researched, and wrote the article.

## Conflict of Interest Statement

The author declares that the research was conducted in the absence of any commercial or financial relationships that could be construed as a potential conflict of interest.

## Footnotes

^1^https://nciterms.nci.nih.gov/.

^2^http://evs.nci.nih.gov/ftp1/NICHD/About.html.

^3^See https://videocast.nih.gov/summary.asp?Live=10941&bhcp=1.

## References

[1] Minnesota Department of Health. The President’s Task Force on Environmental Health Risks and Safety Risks to Children, Activities and Accomplishments (2016). Available from: http://www.health.state.mn.us/divs/eh/children/national.html

[2] H.R. 4365. An Act to Amend the Public Health Service Act with Respect to Children’s Health. Children’s Health Act of 2000, Public Law 106-310, 114 Stat. 1101 (2000). Available from: http://www.gpo.gov/fdsys/pkg/BILLS-106hr4365enr.pdf/BILLS-106hr4365enr.pdf

[3] National Research Council and Institute of Medicine. National Children’s Study Research Plan: A Review. Washington, DC: National Academy Press (2008). 2 p.

[4] Hirschfeld S Songco D Kramer BS Guttmacher AE. National Children’s Study: update in 2010. Mt Sinai J Med (2011) 78(1):119–25. 10.1002/msj.20227 21259268 PMC3247064

[5] Guttmacher AE Hirschfeld S Collins FS The National Children’s Study – a proposed plan. N Engl J Med (2013) 369(20):1873–5. 10.1056/NEJMp1311150 24224620 PMC5101954

[6] Public Law 113-6 Consolidated and Further Continuing Appropriations Act 2013. U.S. Congress, Washington, DC (2013). Available from: https://www.congress.gov/bill/113th-congress/house-bill/933/text?overview=closed&r=1

[7] Duncan GJ Kirkendall NJ Citro CF Panel on the Design of the National Children’s Study and Implications for the Generalizability of Results Committee on National Statistics, Division of Behavioral and Social Sciences and Education, and Board on Children, Youth, and Families, Institute of Medicine. National Research Council and Institute of Medicine, editors. The National Children’s Study 2014: An Assessment. Washington, DC: The National Academies Press (2014). 24945056

[8] Collins F Statement on the National Children’s Study. The NIH Director (2014). Available from: www.nih.gov/about/director/12122014_statement_ACD.htm

[9] Giele JZ Elder GH Jr, editors. Methods of Life Course Research: Qualitative and Quantitative Approaches. SAGE Publications (1998). 22 p.

[10] Bengston VL Allen KR The life course perspective applied to families over time. In: Boss PG Doherty WJ LaRossa R Schumm WR Steinmetz SK, editors. Sourcebook of Family Theories and Methods: A Contextual Approach. New York: Plenum Press (1993). p. 469–99.

[11] Gershon RC Cella D Fox NA Havlik RJ Hendrie HC Wagster MW Assessment of neurological and behavioural function: the NIH Toolbox. Lancet Neurol (2010) 9(2):138–9. 10.1016/S1474-4422(09)70335-7 20129161

[12] Reeve BB Hays RD Bjorner JB Cook KF Crane PK Teresi JA Psychometric evaluation and calibration of health-related quality of life item banks: plans for the patient-reported outcomes measurement information system (PROMIS). Med Care (2007) 45(5 Suppl 1):S22–31. 10.1097/01.mlr.0000250483.85507.04 17443115

[13] Murphy SN Mendis ME Berkowitz DA Kohane I Chueh HC Integration of clinical and genetic data in the i2b2 architecture. AMIA Annu Symp Proc. Washington DC (2006). p. 1040. PMC1839291 17238659

[14] Etzel R Charles M-A Dellarco M Gajeski K Jöckel K-H Hirschfeld S Harmonizing biomarker measurements in longitudinal studies of children’s health and the environment. Biomonitoring (2014) 1:50–62. 10.2478/bimo-2014-0006

[15] Green M Palfrey JS, editors. Bright Futures: Guidelines for Health Supervision of Infants, Children, and Adolescents. Arlington, VA: National Center for Education in Maternal and Child Health (1994).

[16] Hagan JF Shaw JS Duncan PM, editors. Bright Futures: Guidelines for Health Supervision of Infants, Children, and Adolescents. 3rd ed. Elk Grove Village: American Academy of Pediatrics (2008).

[17] World Health Organization. International Classification of Functioning, Disability, and Health: Children and Youth Version ICF-CY. Geneva, Switzerland: WHO (2007).

[18] Data Document Alliance. (2016). Available from: www.ddialliance.org

[19] Clinical Data Interchange Standards Consortium. (2016). Available from: www.cdisc.org

[20] Resolution Adopted by the General Assembly (Without Reference to a Main Committee IA/55/L2) 55/2 United Nations Millennium Declaration. (2000). Available from: www.un.org/en/ga/search/view_doc.asp?symbol=A/RES/55/2

[21] Annul Reports from the Save the Children Foundation. (2016). Available from: http://www.savethechildren.org/site/c.8rKLIXMGIpI4E/b.8663861/k.691F/Online_Library_Archives.htm

[22] Anne E. Casey Kids Count Data Center listings. (2016). Available from: http://datacenter.kidscount.org/publications

[23] Leading Health Indicators from the Healthy People 2020 report. (2016). Available from: https://www.healthypeople.gov/2020/leading-health-indicators/2020-LHI-Topics

