# Supplementary material for: Introduction and Goals for the National Children’s Study
Source: Front Pediatr. 2018 Feb 22;5:240. doi: 10.3389/fped.2017.00240 (PMC5827357; doi:10.3389/fped.2017.00240)
Supplement: Supplementary file 1 [file Table_1.PDF]

Appendix 1: Comparison of Selected Longitudinal Cohort Studies

Study Name	Comments	Year	Approximate Enrollment	Prenatal Enrollment	Representative or Probability Sample	Country
National Child Development Study	England, Scotland and Wales cohort of about 17,000 enrolled during one week in 1958 to examine neonatal death and still birth	1958	17000	No	No	United Kingdom
Northern Finland Birth Cohorts	Birth outcomes	1965	12000	Yes	Modified	Northern Finland
Avon Longitudinal Study of Parents and Children IALSPAC)	Prenatal recruitment Social inequality and the parental life course, Parental employment and the economic security of families, Maternal and paternal health and wellbeing, Child health, development and educational trajectories, Childhood neuropsychiatric,	1991	15000	Yes	No	Avon, England
Czech Early Childhood Study	Five year study associated with ALSPAC and four others as part of the European Longitudinal Study of Pregnancy and Childhood in six countries developed as a World Health Organization initiative	1994	7500	No	No	Czech Republic
Danish National Birth Cohort	Exposures in the period from conception to early childhood - including fetal growth, cell division, and organ functioning - may have long-lasting impact on health and disease susceptibility.	1996	100000	Yes	Modified	Denmark
LISA plus Study	Environmental influences on immune system in Germany	1997	3100	No	No	Germany
Fragile Families and Child Wellbeing	What are the conditions and capabilities of unmarried parents, especially fathers?; (2) What is the nature of the relationships between unmarried parents?; (3) How do children born into these families fare?; and (4) How do policies and environmental conditions affect families and children?	1998	5000	No	Yes	United States

Norwegian Mother and Child Cohort Study	The Norwegian Mother and Child Cohort Study (MoBa) is a study of the causes of disease among mothers and children	1999	90000	Yes	Modified	Norway
National Survey of Child and Adolescent Well Being	Children ages birth to 14 who had contact with the Child Welfare System	1999	6000	No	Yes	United States
Millenium Cohort Study	Instruments available, topics include: parenting; childcare; school choice; child behaviour and cognitive development; child and parental health; parents' employment and education; income and poverty; housing, neighbourhood and residential mobility; and social capital and ethnicity.	2000	19000	No	Yes	United Kingdom
Early Childhood Longitudinal Study-Birth	The Early Childhood Longitudinal Study, Birth Cohort (ECLS-B) was designed to provide policy makers, researchers, child care providers, teachers, and parents with detailed information about children's early life experiences.	2001	14000	No	Yes	United States
Generation R Study	Details on what is collected but no instruments, Topics include: Growth and physical development<br>Behavioral and cognitive development<br>Asthma and atopy<br>Diseases in childhood<br>Health and healthcare	2002	10000	Yes	No	Rotterdam, Netherlands
EDEN	1. biological and clinical aspects: growth and development of the adipose tissue, the masculine genital tract, teeth, as well as immune, respiratory, cardiovascular and metabolic functions 2. behavioral, cognitive and psychomotor development. Multiple births excluded.	2003	2000	Yes	No	France

Amsterdam Born Children and Development- ABCD	Amsterdam Born Children and their Development – study is a long-term, large-scale cohort study, examining the health of children from the very beginning: 8,000 children are followed from the time of their mother's pregnancy until adulthood.	2003	8000	Yes	Modified	Amsterdam, Netherlands
Growing Up Australia	*Growing Up in Australia* is investigating the contribution of children's social, economic and cultural environments to their adjustment and wellbeing. A major aim is to identify policy opportunities for improving support for children and their families and for early intervention and prevention strategies.	2004	10000	No	Yes	Australia
Born in Bradford (BiB)	Questionnaires available, topics include: assess the determinants of childhood and adult disease, asssess the impact of migration, explore the influences of pregnancy and child birth on subsequent health, generate and test hypotheses that have the potential to improve health for some of the most disadvantaged within our society	2007	13500	Yes	No	Bradford, England
Cork Ireland Baseline Study	Initially focus on three main research questions: the effects of poor growth in the womb, the incidence and prevalence of food allergy and eczema in early childhood and the incidence and effects of maternal and infant vitamin D status on the growth and health of Irish children.	2008	3000	No	No	Ireland
Growing Up in New Zealand	Health and wellbeing, psychosocial and cognitive development, education, family, culture and identity, societal context and neighborhood environment	2009	7000	Yes	Modified	New Zealand

ELFE	Demography-family; Socialization-education; Economy-poverty; Feeding-nutrition; Psychomotor development and mental health; Use of health care; Respiratory diseases, asthma and allergies; Accidents and traumas; Chemical exposures; Physical exposures; Environmental contaminations (air, water)	2011	20000	No	Yes	France
Japan Environment and Children's Study	The Japan Environment and Children's Study (JECS), a birth cohort study involving 100,000 parent-child pairs, was launched in 2011 in order to evaluate the impact of various environmental factors on children's health and development.	2011	100000	Yes	No	Japan
Life Study	Prenatal recruitment Social inequality and the parental life course, Parental employment and the economic secutiry of families, Maternal and paternal health and wellbeing, Child health, development and educational trajectories, Childhood neuropsychiatric, developmental and neurological disorders, growth in infancy, childhood obesity, nutrition and physical activity, environment and health	2014	80000	Yes	Yes	United Kingdom
